# Future Trends in Nebulized Therapies for Pulmonary Disease

**DOI:** 10.3390/jpm10020037

**Published:** 2020-05-10

**Authors:** Sean D. McCarthy, Héctor E. González, Brendan D. Higgins

**Affiliations:** 1 Anaesthesia, School of Medicine, National University of Ireland Galway, H91 TK33 Galway, Ireland; sean.mccarthy@nuigalway.ie (S.D.M.); h.gonzalez2@nuigalway.ie (H.E.G.); 2 Lung Biology Group, Regenerative Medicine Institute (REMEDI) at CÚRAM Centre for Research in Medical Devices, National University of Ireland Galway, H91 TK33 Galway, Ireland; 3 Physiology, School of Medicine, National University of Ireland Galway, H91 TK33 Galway, Ireland

**Keywords:** aerosol therapy, ARDS, COPD, intensive care unit, jet nebulizer, mechanical ventilation, sepsis, vibrating mesh nebulizer, nebulization

## Abstract

Aerosol therapy is a key modality for drug delivery to the lungs of respiratory disease patients. Aerosol therapy improves therapeutic effects by directly targeting diseased lung regions for rapid onset of action, requiring smaller doses than oral or intravenous delivery and minimizing systemic side effects. In order to optimize treatment of critically ill patients, the efficacy of aerosol therapy depends on lung morphology, breathing patterns, aerosol droplet characteristics, disease, mechanical ventilation, pharmacokinetics, and the pharmacodynamics of cell-drug interactions. While aerosol characteristics are influenced by drug formulations and device mechanisms, most other factors are reliant on individual patient variables. This has led to increased efforts towards more personalized therapeutic approaches to optimize pulmonary drug delivery and improve selection of effective drug types for individual patients. Vibrating mesh nebulizers (VMN) are the dominant device in clinical trials involving mechanical ventilation and emerging drugs. In this review, we consider the use of VMN during mechanical ventilation in intensive care units. We aim to link VMN fundamentals to applications in mechanically ventilated patients and look to the future use of VMN in emerging personalized therapeutic drugs.

## 1. Introduction

Over the past 27 years since the commercial release of the first ultrasonic mesh nebulizer [1], this method of aerosol generation has become well established in respiratory drug delivery. Compared to pressurized metered dose inhalers (pMDI) and dry powder inhalers (DPI), which are highly portable and do not require electrical power or compressed gas to drive nebulization, nebulizers have tended to be utilized primarily in hospitals, where the longer timeframe required to deliver drugs is also less demanding. However, as nebulization technologies have developed and improved over the past quarter of a century, this has expanded the reach of nebulizers not just in clinical settings but also in the home, where pMDI and DPI previously dominated.

As new nebulization technologies emerge, the role of nebulizers in respiratory drug delivery continues to evolve. Adaptable nebulizer technology is ideal for combination in aerosol delivery systems where the objective is to augment lung dose by controlling, and possibly calculating and adapting to, a patient’s breathing patterns. The role of nebulizers in treating respiratory disease, and symptoms of disease is the subject of numerous recent reviews, including in acute respiratory distress syndrome (ARDS) [2,3], chronic obstructive pulmonary disorder (COPD) [4,5], cystic fibrosis (CF) [6,7], ventilator-associated pneumonia (VAP) [8,9], dyspnea [10], and acute asthma [11], among others.

Features of pulmonary physiology, such as a large surface area, a thin alveolar-blood barrier, along with the combined advantages of low enzymatic activity and first-pass metabolism, present the possibility of attaining high bioavailability when aerosolized drugs are delivered via the lungs [12]. The primary aim of inhalational aerosol therapy is to deliver high concentrations of drugs either directly to lung tissue to target respiratory diseases [13] or via the lungs as a route to target systemic diseases, such as cancer or diabetes [14,15]. Directly targeted pulmonary drug delivery has crucial benefits over systemic administration, such as more rapid onset of action, increased therapeutic effects, and reduced systemic side effects, as the required lung dosage can be achieved with lower doses as well as the opportunity to repurpose existing drugs [16] leading to increased interest in the field of aerosolized drug therapy in intensive care units (ICU).

## 2. Development of Nebulizers

The potential of pulmonary drug delivery to treat respiratory diseases has been recognized for millennia with accounts of therapeutic drug delivery via aerosols dating back to ancient Egypt [17]. Conventional inhalation therapy can be traced to the invention of glass bulb nebulizers in the 19 century, considered true precursors of contemporary inhaled and aerosol therapy [18].

The primary feature of nebulizers is the conversion of liquid drug formulations by either mechanical or thermal energy into inhalable emitted aerosols that can be deposited in the distal lungs due to their advantageous droplet size and output rates [19,20,21,22]. Nebulizers are generally used to deliver inhalational therapy in patient populations that cannot effectively use pMDI or DPI, such as pediatric, geriatric, and critically ill patients where difficulties with synchronization of inhalation and device actuation are likely (issues relate to coordination, age, cognitive status, dexterity, and strength), leading to reduced lung delivery [23,24,25]. Nebulizers are vitally important to deliver inhalational therapy in either invasive or non-invasive mechanical ventilation in non-ambulatory settings [26,27,28].

The deterioration of lung function in disease as well as the need to treat acute exacerbations results in patients often requiring treatment in acute care settings and the delivery of aerosolized medications, such as antibiotics, bronchodilators, and inhaled corticosteroids. There are strong trends particularly in high-income countries towards increased admissions to and length of stay in ICU for pediatric [29,30] and especially geriatric patients with sepsis, respiratory disease, or nosocomial acquired conditions [31,32,33]. Diseases requiring inhalation therapies include asthma, bronchiectasis, COPD, CF, pneumonia, and ARDS [34,35,36]. Common nebulized medications used to treat these conditions include antibiotics [37,38], various bronchodilators, such as albuterol sulfate [39] and ipratropium bromide [40], and corticosteroids, such as budesonide [41].

An MDI with a spacer may be used in alert pediatric patients and during mechanical ventilation, though typical doses are limited to <0.5 mg. Whereas drug doses of >100 mg can be delivered with most nebulizers. Another important consideration favoring the use of nebulizers is that many newly developed medications and most biologics are first produced as liquid formulations which favors nebulizer delivery [20]. Another relatively new aerosol platform with similar technique requirements as MDI is the soft mist inhaler (SMI) (Respimat^®^). This inhaler delivers a fixed-dose combination of a long-acting muscarinic antagonist (LAMA; tiotropium bromide) and a long-acting β2-adrenergic agonist (LABA; olodaterol) which is operated by a patient actuated spring mechanism causing the collision of two accelerating aerosol jets via two separate nozzles [42]. Respimat SMI deposition to the whole lung and the peripheral airways is higher than MDI or DPI [43]. The combination of two bronchodilators of different action durations and mechanisms may increase the degree of bronchodilation. There are few studies showing the use of this device in mechanically ventilated patients though it should be beneficial in weaning COPD patients from ventilators [44].

As refinements of existing devices continue, but perhaps the pace of development slows, new mechanisms of nebulization emerge. The nascent technology of surface acoustic waves offers a new aerosol generating prospect. Surface acoustic wave nebulizers (SAWN) produce relatively short wavelength but high frequency (10 to 100 MHz) sound waves which travel along the surface of a liquid to generate aerosols. While there are currently few in vivo human studies, the potential of SAWN has been assessed for aerosol generation of peptides and demonstrated retained monoclonal antibody and antimicrobial integrity as well as bioactivity activity and appropriate particle size for deep lung penetration [45,46]. While SAWN offer similar benefits compared to vibrating mesh nebulizers (VMN) in terms of reduced shear stress, they may also prove less susceptible to clogging, particularly when particle suspensions or larger macromolecules are used.

When considering these different devices, the need to minimize side-effects as well as efficient delivery is vital to provide physiologically effective doses in infants, the elderly, and critically ill ventilated patients. It is essential that wasteful or inconsistent delivery of aerosolized emerging drugs or biological therapies be minimized so that the full promise of these expensively developed therapies is realized in clinical trials.

## 3. Vibrating Mesh Nebulizers

Patients with severe exacerbations or respiratory conditions that require acute intensive care may also have additional support requirements such as invasive mechanical ventilation; therefore, nebulizers have potentially unique clinical effectiveness profiles in this patient population. Aerosol generating devices used to generate therapeutic aerosols for ventilated patients include MDI, DPI, slow mist inhalers, jet nebulizers (JN), ultrasonic nebulizers, and vibrating mesh nebulizers (VMN), and there are strengths and limitations to the use of each depending on patient requirements [47].

JN are the standard and lowest cost device for inhaled medications, though they require large flows of external medical gas to generate aerosols whereas MDI and VMN do not [47] and are comparatively wasteful resulting in limited dose [48,49]. These are not pressing concerns in hospitalized patients receiving highly potent (microgram doses), inexpensive bronchodilators where doses can be titrated to account for typically rapid effects at the bedside. JN have maintained a relatively similar performance standard since their introduction. For possibly less potent but more expensive drugs, such as emerging therapeutics or antibiotics requiring larger doses and longer response times, the less apparent therapeutic effects will not be easily judged [48].

VMN are quiet and portable which confers benefits in both home and ICU settings [47]. VMN are associated with low residual drug volumes [22,50] and greater inhaled mass in models of in vitro invasive ventilation [51]. Aerosol particles in the size range of ~2–6 μm are suited to maximal tracheobronchial deposition while smaller particles within the range ~0.5–3 μm are maximally suited to alveolar deposition in the alveoli [2]. The small particle size emitted by VMN is associated with less impactful aerosol loss in ventilator breathing circuits and airways. As VMN do not require added gas flow into the ventilator circuit to operate, they do not dilute the aerosol or detrimentally alter pressures in the circuit or the volumes delivered [52].

The continuing development of VMN has seen increased application and research of aerosol therapy in critical care settings because of the potential of these devices to reliably generate aerosol particle sizes which are considered optimal for deep lung penetration [20,26,53].

### 3.1. Advantages of Vibrating Mesh Nebulizers in Mechanical Ventilation

While the nebulization market is dominated by JN, in part due to being packaged together with existing drugs, VMN are now the leading choice in sponsored clinical trials in both the EU and US, particularly trials involving new, expensive or niche therapeutics. Aerogen Ltd. VMN technologies are the dominant device platform for mechanical ventilation trials [1]. This platform has been shown to retain the bioactivity of antibiotics [51], gene vectors [54], inhaled vaccines [55], or proteins [56,57]. The clinical effectiveness of VMN has been demonstrated in outcomes such as shorter ICU stays compared to MDI and JN in mechanically ventilated asthmatic patients [58]. VMN deliver significantly increased total inhalable dose compared to JN in mechanical ventilation [59]. Indeed within the current COVID-19 pandemic, the recommended method of aerosol delivery for ventilated patients is via VMN with an additional filter placed at the expiratory port during nebulization to limit fugitive nosocomial transmission [60].

Another key advantage of VMN is the minimal residual volumes of usually <10% compared with both ultrasonic nebulizers (up to 30%) and JN (up to 50%) [22,61] mainly due to the absence of baffles which tend to trap drugs within the medication chamber, reducing the delivered dose. This design advantage has facilitated both reductions in fill volumes (almost five-fold) for the I-neb AAD System breath-activated mesh nebulizer compared to a JN [62] and, in vivo, a tripling of mean lung deposition by the MicroAir NE-U22 mesh nebulizer as a percent of volume fill compared to the same JN [63]. The absence of baffles also means drugs need only pass through VMN devices once to generate aerosol rather than being recycled numerous times, which reduces shear stress and the probability of damaging the drug, additionally there is little evaporative loss or cooling of the drug, all of which are significant for new bioactive therapies [64]. Some VMN also integrate breath-actuated features to stop wasteful aerosol production during exhalation or draw extra air into the device to reduce wastage and improve drug delivery [65,66,67]. The potential for adverse effects with off-label or new drugs and the likelihood that novel personalized medications will be relatively expensive during development favors the use of nebulizers which provide dose reproducibility to ensure economic and safer drug delivery. Breath-activated VMN systems such as AKITA^2^ APIXNEB nebulizer systems [68], Pneuma Inhaler [69], I-neb AAD System [70], or the Pulmonary Drug Delivery System [71] offer potential for more personalized drug delivery which should all eventually fully integrate into mechanical ventilation circuits.

An important factor for future utilization of VMN is the capability to generate specifically tailored aerosols via re-engineered mesh technologies which will accommodate new drugs or personalized therapies packaged or developed with VMN in mind [72]. Developments of VMN technology continue with multiple new brands of device and refinements of technology. Aerogen have developed a new mesh technology, photo defined aperture plate or PDAP, a two-layer nebulizer with 15,600 preformed holes compared to their Pro device with 1000 holes. The PDAP nebulizer generates low droplet sizes with faster flow rates [50]. Refinements of this nature have the potential to improve patient outcomes by improving drug deposition in the lower airways whilst also reducing treatment times.

In terms of disadvantages of VMN, as nebulizer delivery times tend to be longer compared with other devices [51], it is more likely that aerosol drug therapy will be disrupted by other necessary clinical procedures [73]. There is also a tendency for more viscous or suspension type formulations to clog the apertures of mesh plates. Thorough cleaning is required to maintain function of VMN, increasing labor requirements, nonetheless nebulization time can increase with frequent use necessitating replacement [64] which is not ideal as VMN have greater costs up-front [66].

### 3.2. Factors Affecting Aerosol Delivery

Though mechanically ventilated patients are routinely treated using inhalation aerosols, there are inherent invasive delivery challenges. Patient related factors which influence drug delivery-include age, sex, lung regions affected and how severely the particular disease has affected the respiratory system [74,75,76]. This has led to a trend towards personalized therapeutic approaches which target optimized pulmonary delivery and selection of the most effective type of drug for individual patients [75]. Mechanical ventilation in the most critically ill patients poses additional complications including the interface with the patient (e.g., endotracheal tube or tracheostomy tube [77,78]), the gas pressures and flow characteristics within the breathing circuit, and not least, ventilator settings. Additionally, to achieve optimal therapeutic benefits, several factors influence the characteristics of aerosol particles emitted by nebulizers which determine drug distribution in the lungs. Such factors include drug formulation (viscosity, delivery platforms), nebulizer performance (particle size and shape, fine particle fraction (% <5 μm) and device type. Comprehensive reviews of the various factors influencing aerosol deposition during mechanical ventilation beyond the scope of the present review are available [12,79].

## 4. Drug Therapy in Critically Ill Patients

### 4.1. Pulmonary Drug Formulations

The combination of pulmonary epithelium targeted drug delivery, new devices and new delivery technologies suggests an exciting development phase for emerging pharmaceuticals. New delivery technologies such as nanoparticles, microemulsions, and lipid-based carriers are increasingly being designed for pulmonary treatments. Innovative delivery platforms like these can be used not only to target pulmonary disease, but also to deliver drugs systemically through the large surface area of the pulmonary epithelium facilitating relatively straightforward entry to the blood stream [4,80,81]. VMN, such as the Aerogen Pro or AeroProbe, have proven compatible with delivery of both lipid-based carriers [82,83] and nanoparticles [84,85].

Liposomes are a multipurpose platform allowing delivery of nucleic acids, peptides and small molecule therapies, with the added benefit of encapsulating hydrophilic drugs within an aqueous core and integrating lipophilic compounds within the phospholipid bilayers. The components of phospholipid-based liposomes are also biocompatible and biodegradable within lungs [81,86]. The use of reformulation of existing drugs for asthma, COPD, CF and infections into a range of different doses using liposomes and lipid microparticles for pulmonary treatments is occurring and as new therapeutic molecules and gene therapy continue to develop these platforms offer a tested delivery methodology [86,87]. Aerosolized liposomes and lipid-based microparticles also provide sustained delivery and superior pharmacokinetics profile within the pulmonary system, protection against inherent enzymatic degradation within the lungs and benefits in pulmonary disease treatment. In addition, lipid-based delivery may reduce the dosing frequency which will improve therapeutic outcomes. While lipid-based carriers can reduce inflammation, toxicity or adverse effects of drugs [86,88].

Various types of nanoparticles may also provide alternative formulations for nebulizers to enhance pharmacokinetics of key antibiotics, anti-inflammatory agents and bronchodilators. Nanoparticles have potential to improve pulmonary and systemic drug therapy through controlled and precise delivery, with improved bioavailability [81,89]. The smaller particle size of nanoparticle confers several advantages over larger particle platforms including greater concentration of drug molecules on the surface rather than particle interior and decreased time to dissolution. Additionally, the enhanced solubility of nanoparticles compared to larger particles will increase the bioavailability of even poorly soluble hydrophobic drugs and facilitate higher doses [90,91,92,93].

As new therapies emerge it is doubtful whether all the new technology formulations will be compatible with all new drugs or dosing indications. Therefore, it will be necessary to select delivery platforms considering the physicochemical properties of the drugs and the biological and pharmaceutical mechanisms influencing delivery in combination with VMN.

### 4.2. Personalized Medicine

Advances in genomic information and subsequent utilization in medicine is strongly linked to the rate advancement in genomic technology and science. However, the downstream applications of such personalized medicine are numerous and exciting and a number of conditions have progressed through path of gene discovery, disease research and development of a novel therapeutics, leading to clinical trials [94]. Although the era of personalized medicine is only in its infancy, it will be a critical aspect of future combinations of science and medicine. In 2016, an integrative genomics initiative was established to change drug discovery and development by bringing novel insights into the pathophysiology of diseases and to identify new drug targets, enhancing patient selection for clinical trials and matching patients to potential therapies to maximize benefits [95]. The value of a more personalized approach to therapy regimens with respect to dose, treatment interval and potential self-administration would be a significant advance in medicine practices [96]. Aerosolization of therapeutic agents and delivery via nebulizer can have a significant impact on this emerging era of medicine.

### 4.3. Acute Respiratory Distress Syndrome

ARDS is a severe lung injury syndrome with a mortality rate of ~40%, characterized by hypoxemia, pulmonary edema and loss of lung compliance [97,98]. ARDS commonly occurs as a result of an inflammatory response triggered by local (pneumonia) or systemic (sepsis) infection [99]. Ten percent of all ICU patients develop ARDS, and the incidence increases when mechanical ventilation is required [100]. No specific treatment exists for ARDS, so protective lung ventilation strategies, fluid support and early broad-spectrum antibiotics are the chosen adjunctive treatments. Patients with severe hypoxemia due to pneumonia or ARDS poorly tolerate optimum nebulization settings when ventilated and require deeper sedation and muscle relaxation, which extends the duration of mechanical ventilation and ICU stay. Glucocorticoids, antioxidants, surfactants, inhaled nitric oxide, protease inhibitors, and a variety of other anti-inflammatory treatments have been tested in clinical trials [101]. However, none have proven significantly beneficial, either lacking therapeutic effect or displaying side-effects when administered via existing parenteral and systemic methods. Therapeutically significant delivery of these aerosols to the lungs may have been an additional challenge which may have hindered the potential of some of these trialed modalities, yet nebulization offers a less intrusive yet penetrative delivery to target regions of the lungs.

Bronchodilators are routinely nebulized into ventilated patients to improve the air flow to the lungs. The most used is albuterol sulfate which has also been shown to improve fluid clearance, a critical aspect of ARDS treatment. Several preclinical ARDS model studies support this idea [102,103] but clinical trials have not observed significant improvements in patients [104], though they suggest early administration and co-administration with glucocorticoids may prevent lung damage in ARDS development. Aside from the ICU stay, most ARDS survivors need personalized bronchodilator treatment after hospital discharge due to reduced respiratory function and nebulization can offer an easy delivery option for treatment at home.

Inhaled glucocorticoids present a significant opportunity to manage the developing inflammatory process in ARDS patients. Preclinical studies have demonstrated improvements in indices of histological injury in a porcine ARDS model [105], while significantly reduced inflammatory markers and improved oxygenation were found after administration of nebulized budesonide in acute lung injury (ALI) patients [106]. In a further pharmacologic attempt to attenuate inflammation in ARDS, nebulized heparin has attenuated inflammation and reduced damage in a preclinical model of ALI [107]. Recently, nebulized heparin, in combination with N-acetylcysteine and albuterol sulfate, reduced the time under ventilation for patients with inhalation injury [108].

New nebulized pharmacologic approaches are developing in ARDS therapy. Mucolytic agents reduce the viscosity of mucus, normally difficult to eliminate in ventilated patients due to the lack of coughing, facilitating pulmonary clearance. N-acetylcysteine is the most prescribed mucolytic but clinical administration by nebulization is not well studied. Another mucolytic, hypertonic saline has been shown to attenuate the severity of ALI when nebulized, by reducing inflammatory cytokine production [7,109]. The potential for a more personalized approach to treatment is evident from studies such as the demonstration that a phosphorylation resistant IκBα super-repressor plasmid was nebulized into to an endotoxin-induced lung injury model. The therapeutic plasmid was seen to attenuate parameters including bronchoalveolar lavage detected neutrophilia, interleukin-6 and cytokine-induced neutrophil chemoattractant-1 levels, total protein content, as well as histologic indices of injury [54]. Such approaches lay vital groundwork for a more personalized therapeutic approach to ALI/ARDS whereby specific therapeutic candidates could be utilized to ameliorate specific processes associated with a disease or syndrome. ARDS patients also present disturbances in the composition and function of surfactant responsible of maintaining alveolar surface tension and facilitation of the lung immune response [110]. The exogenous administration of surfactant has been proven in premature pediatric patients but while aerosolized surfactant was well tolerated at the doses administered, it did not significantly improve physiological parameters in patients with sepsis induced ARDS. New drug formulations or nebulizer device combinations may have potential to improve delivery [111]. A number of in vitro, preclinical, and clinical studies had shown potential benefits from statin administration in ARDS and potentially other lung diseases by attenuating inflammation and protecting respiratory function [112]. Disappointingly, two recent randomized clinical trials (SAILS (rosuvastatin) and HARP-2 (simvastatin)) failed to demonstrate improved survival or ventilator-free days of ARDS patients treated with high-dose enteral statins. Indeed, safety concerns were flagged in rosuvastatin-treated patients, with increased hepatic and renal organ dysfunction [113]. These adverse systemic effects were not reported in the HARP-2 trial but the authors acknowledge the high-dose used may have contributed negatively to overall outcomes and currently simvastatin is not recommended in ARDS [114]. Retrospective analysis of the HARP-2 trial demonstrated some benefits of statins may accrue in the hyperinflammatory subphenotype cohort compared to a more heterogenous ARDS patients group [115]. Simvastatin has potential as an anti-inflammatory, antioxidant, and muco-inhibitory drug to the airways and lung. Emerging aerosolized formulations such as lipo-core nanoparticles [116] could permit both customization and optimization of the lung targeted aerosols to reinvigorate the potential benefits of statins, such as rapid onset, lower adverse side effects, and improved drug stability [117,118].

## 5. Aerosolized Therapies

Multi-drug resistant infections are a growing worldwide health problem necessitating an urgent need to produce alternative antimicrobial agents. Also, as populations of wealthier countries age, more patients with various pulmonary conditions are hospitalized or require critical care in ICU.

### 5.1. Antibiotics

Nebulized antimicrobials have become a key therapy to eradicate and suppress *Pseudomonas aeruginosa* colonization in CF patients [119]. They provide decreased toxicity and increased efficacy by targeted delivery, however, increasing treatment times has the potential for contamination and requires additional maintenance [120]. Nebulized colistin, gentamicin, and tobramycin therapy is also recommended in non-CF bronchiectasis [121] based on studies demonstrating a reduction in bacterial density although verified long-term improvements in lung function remain elusive [122]. The emergence of multi drug resistant bacteria, often requiring the use of nephrotoxic antimicrobials, such as colistin, has created an additional role for nebulized therapy. Nebulized antimicrobials have been used as monotherapy or in conjunction with parenteral antimicrobials to treat respiratory tract infections. Studies have demonstrated that nebulized colistin is associated with microbiological eradication of pneumonia, however, there are conflicting reports on the impact of nebulization on clinical outcomes and mortality [123]. A recent retrospective observational study by Leache et al. (2020) compared the use of systemic antimicrobials to systemic antimicrobials with adjunctive nebulized antimicrobials for pneumonia or tracheobronchitis. The combination of systemic and nebulized antimicrobials was associated with enhanced clinical resolution without increased renal toxicity [124]. Another recent single-arm clinical trial evaluated clinical outcomes of nebulized (off-label) plus intravenous vancomycin antibiotic in mechanically ventilated patients with MRSA pneumonia. Ventilator-associated pneumonia which may develop into ARDS, is a prevalent nosocomial infection in the ICU, with multidrug-resistant bacteria (e.g., methicillin-resistant *Staphylococcus aureus* (MRSA)) a frequent cause. MRSA nosocomial pneumonia is often treated with systemic vancomycin or linezolid, but therapeutic benefits are inadequate, with poor lung penetration in the critically ill, while prolonged prescription of vancomycin is associated with significant nephrotoxicity. Despite the small sample size, nebulized vancomycin showed increased lung dosing, effective microbiological eradication and no additional side-effects [125].

Nebulized antimicrobial therapy has also been assessed for the treatment and prevention of fungal infection. Nebulized liposomal amphotericin B has been investigated for prophylactic therapy for invasive pulmonary aspergillosis in chemotherapy induced neutropenia. The toxicity and adverse events associated with systemic liposomal amphotericin limit its use making inhaled therapy a preferable alternative. A randomized, double-blind, placebo-controlled trial demonstrated nebulization of liposomal amphotericin B significantly reduced the incidence of invasive pulmonary aspergillosis in adult patients with chemotherapy induced neutropenia compared to placebo with no systemic toxicity noted [126]. New antibiotics are continually undergoing testing, of particular interest is the development of non-antibiotic antimicrobials, which may allow treatment of antibiotic resistant organisms [127]. Some studies have also demonstrated that it is possible to generate a respirable aerosol of antimicrobial peptides (AMPs) or prodrug AMPs. The AMP prodrug and its active peptide component were both unchanged after VMN and maintained their levels of antimicrobial activity against the most common CF pathogen, *P. aeruginosa* [56].

### 5.2. Vaccines and Gene Therapy

Aerosolized vaccines have the potential to be used as a needle-free alternative for several diseases. An aerosolized measles program was introduced in Mexico as early as the 1980s [128]. Improved antibody booster response to an aerosolized measles vaccine has been shown compared to injection. This advantage is maintained with aerosolized doses less than or equal to one-fifth of the usual injected doses [129]. However, other reports on the efficacy of VMN delivered measles vaccine in children are inconsistent, reporting aerosolized vaccine to be inferior to the subcutaneous vaccine in terms of seropositivity [130,131], though the vaccine might be effective in older children than those studied and a second dose of measles vaccine may be necessary.

DNA vaccines can be produced efficiently and are inexpensive [132]. Administration of DNA vaccines to the respiratory mucosa is an emerging field but nebulization of DNA to achieve uptake by lung cells offers a valuable methodology [133]. Rajapaksa et al. (2014) reported successful inhalation delivery of a plasmid DNA vaccine in a large animal model using a SAWN device [134]. It is worth noting that cationic liposomes and plasmid DNA complexes have been the predominant alternative to viral vectors utilized in studies of inhaled gene delivery to the respiratory tract [135,136]. A recent phase 2b trial implemented a gene-liposome complex aerosolized through a Trudell AeroEclipse II device [137]. Repeated administration of the CFTR gene therapy formulation for one year, was associated with a significant improvement in lung function.

Various mRNA vaccine platforms have been developed recently which have the potential to induce the body to make its own treatment. This approach has been validated in studies of immunogenicity and efficacy showing the potential to be safer than other vaccines [138,139]. Personalized immunotherapy which has the potential to be tailor-made to match the genetic profile of a patient’s cancer or the potential of mRNA vaccines against influenza or Zika virus offer a promising [140,141]. A comparison of direct intratracheal instillation versus nebulized delivery of in vitro transcribed mRNA to the lung did not affect transfection efficiency, though a slight reduction in transfection efficiency was observed, this was remedied by increasing the concentration of transfection reagent [142]. Similarly, nebulization of miRNA nanoparticle complexes resulted in successful deposition in secondary bronchi and bronchioles upon inhalation, while VMN did not affect physicochemical properties or transfection efficiency of the complexes [143]. New biotech companies, such as Factor Bioscience, are pioneering nucleic-acid and cell-based technologies to advance the study and treatment of disease which are undergoing nebulization testing. As an alternative to maintaining the integrity of nebulized nucleic acids, some RNA-Seq experiments have implemented nebulization as a means to shear and fragment cDNA strands prior to sequencing [144,145]. One of the biggest challenges to nucleic acid and cell-based therapies remains reaching the correct tissues and target cells. For pathologies where the airway is the target; aerosolized delivery offers significant potential, however, this non-invasive and potentially safer targeted administration has not yet been fully utilized.

### 5.3. Heparin and Mucolytics

Heparin is a polysulfated polysaccharide that is used clinically as an anticoagulant but also possesses potent antiprotease activity against serine proteases as well as anti-inflammatory properties [146]. Clinical trials of patients with smoke inhalation trauma suggest that local treatment with both instilled and nebulized heparin may have beneficial effects [147,148]. However, a 2016 meta-analysis provided no convincing evidence for benefit of heparin nebulization in intubated and ventilated ICU patients [149], while others suggest that nebulized heparin, possibly in combination with mucolytics such as *N*-acetylcysteine (NAC) may be beneficial in reducing mechanical ventilation duration but has no survival benefit in ARDS patients [108]. Heparin combined with NAC showed significant improvement in airway edema and a resolution of soot in patients with inhalational smoke-induced lung injury and mild-to-severe lung injury scores [150]. A recent case study described the use of nebulized NAC as a life-saving mucolytic following failed conventional mucolytic therapy [151]. As a treatment for aspergillus, NAC can be nebulized as a 20% solution (Mucomyst^®^) via the Pari Sprint device often in combination with a bronchodilator to offset the possibility of NAC-induced bronchospasm [152]. Nebulized NAC appears to have a different mechanism of action from oral NAC, with nebulization the preferred route of administration if reduced viscosity of pulmonary secretions is the goal [153]. Pulmonary arterial hypertension (PAH) can affect infants, children, and adults and is characterized by pulmonary arteriolar constriction, pulmonary vasculature remodeling and consequent elevation in pulmonary vascular resistance leading to increased right heart strain and ultimately right-sided heart failure. Using an Aerogen Solo VMN to deliver treprostinil in a variety of simulated conditions and in vitro patient models including an infant, pediatric, and adult models found that the VMN is a suitable alternative for inhaled treprostinil delivery in both mechanical ventilation and spontaneous breathing compared to the supplied ultrasonic nebulizer based system [154].

### 5.4. Mesenchymal Stem Cells

In the last decade a promising new therapeutic resource has appeared in the field of lung therapy. The immune modulatory capacity of stem cells, the low immunogenic reaction and the improvement in their isolation, culture and expansion has made them an excellent candidate for lung injury treatment. Several in vitro and preclinical studies have revealed the potential of stem cells, especially mesenchymal stem cells (MSCs), in the treatment of different pulmonary diseases. It has been demonstrated that MSCs reduce bacterial load and reduce inflammatory markers in pneumonia models [155,156]. Several studies have demonstrated the capacity of MSCs to reduce the inflammatory response characteristic of asthma patients [157] and their regenerative properties in fibrotic diseases such as COPD [158,159]. Several clinical trials are underway in order to prove the safety and efficacy of these treatments [160,161,162,163]. The mechanism of action of MSCs is still under investigation but insight into these cells has improved recently. MSCs exert their influence by cell contact and the secretion of paracrine factors. These factors include AMPs, anti-inflammatory cytokines and growth factors that have proved pivotal in MSC effects [153,164,165,166,167]. Despite the promising vista of MSCs as a viable treatment in several lung diseases, there is still room for improvement. The safety of the administration of these cells has been reported for several preclinical and clinical trials [162,168], but direct administration of MSCs risks not targeting the affected organ or losing therapeutic effect. In the lung, the direct administration of MSCs suspensions could produce a tissue injury due to the direct administration of liquid to the lung where fluid accumulation is already an issue, thus further exacerbating the disease. Nebulization of cells or their biological product could solve some of these problems. Kim et al. (2016) showed MSCs retain engraftment potential after been nebulized [169], while McCarthy et al. (2019) showed that VMN delivered MSC-derived conditioned media retain their antibacterial capacity [170], probably by maintenance of AMPs. Supporting this hypothesis, Forde et al. showed that VMN delivery of AMPs does not affect their function [56], while Casciaro et al. (2019) demonstrated that JN emitted esculentin-1a-derived antimicrobial peptides did not lose the therapeutic effect compared with the intra-tracheal installation [171]. There is some pioneering research supporting the idea of the maintenance of integrity and function of small bioactive molecules after nebulization, like those associated with the MSC secretome. Using a non-commercial nebulizer based on eFlow technology (PARI Pharma GmbH) a specially optimized aerosol of IgG, IgA, and IgAM did not affect activity of these molecules and they could be detected 72 h after nebulization in bronchoalveolar lavage samples in a *Streptococcus* pneumonia model in mice [172]. Other molecules such as interferon-gamma (INF-γ), a macrophage stimulator factor, also produced by MSCs, have been proven retain bioactivity after nebulization. Sweeney et al. (2019) showed that VMN produced aerosol of purified INF-γ retained molecule stability and activity in an in vitro study with the THP-1 macrophage cell line [50]. Similarly, nebulized INF-γ has greater effect compared to parenteral delivery in idiopathic pulmonary fibrosis patients in a controlled clinical trial [173,174]. Similar fibrotic pathways are activated in fibrosis and COPD, however, the combination of the therapeutic potential of stem cells and the delivery advantages of nebulization offers a bright future for lung disease treatments.

### 5.5. Antibodies

The pulmonary delivery of monoclonal antibodies is an attractive proposition for the treatment of pulmonary diseases and some of the earliest studies showed viable and beneficial effects [175,176]. However, formulations need to be optimized for each antibody’s properties and its paired aerosol device’s specification [177], with VMN recommended as the most appropriate device to achieve safe, large lung doses and long-term retention [178]. The human GSK1995057 antibody directed against the TNFR1 receptor was nebulized into 37 healthy volunteers challenged with inhaled endotoxin. Nebulized GSK1995057 attenuated pulmonary neutrophilia, inflammatory cytokine release, and signs of endothelial injury in bronchoalveolar lavage and serum samples [179]. Preclinical studies in aerosolized antibody administration are ongoing, for example a recent study showed that nebulized immunoglobulins could be delivered while maintaining protein integrity in both rat and non-human primate lungs from the bronchi to the alveoli [172].

### 5.6. Short- and Long-Acting Adrenergic and Muscarinic Agonists

Over the last decade inhaled therapies for COPD and asthma have come to the fore in clinics to harness improvements in therapeutic effect. Different delivery methods have been utilized, from pMDI, DPI, SMI, and nebulizers. As detailed earlier, nebulization offers advantages over other delivery devices in ambulatory and critically ill patients while allowing compatible formulations to maintain their therapeutic effect [180]. Established treatment for COPD involve short-acting β2 agonists (SABA), short-acting muscarinic antagonists (SAMA), long-acting β2 agonists (LABA), and long-acting muscarinic antagonists (LAMA). LABA and LAMA, and combinations of both, are more commonly used based on better outcomes for airflow, reduction of air trapping, and improvement of exercise intolerance over long treatment periods [181]. Some recent SAMA studies showed nebulized ipratropium bromide reduced tracheal and bronchial secretions during bronchoscopy, improving patient comfort [182]. Similarly, emerging inhaled pharmacologic therapies, such as RPL554 (dual phosphodiesterase 3 and 4 inhibitor), have been investigated in clinical trials for short-term bronchodilator effects in COPD patients, but conclusive results have yet to emerge [183]. Outside of COPD, nebulized ipratropium bromide has been proven to have a bronchodilatory effect on patients with familial dysautonomia, improving outcome [184]. Based on the increased use of LAMA and LABA therapies, several studies have been conducted to assure the efficacy and safety of the delivery of these therapies using nebulization. Some in vitro studies support the delivery efficacy of this method, including the eFlow CS generates glycopyrrolate aerosols with high delivered dose, short treatment time, and small droplet size with narrow size distribution suitable for central and peripheral airway deposition. The unit dose per vial mitigates medication misuse and ensures dose uniformity [185]. Nebulized glycopyrronium bromide produced similar bronchodilation but lower systemic levels of drug than delivery by DPI, also, patients reported lower number of adverse effects using the eFlow CS nebulizer [186]. A large study divided into 6 clinical trials assessed the safety and efficacy of nebulized glycopyrrolate in the long-term treatment of COPD. Some of the results of these clinical trials have been published recently, showing no adverse effects in the patients, and improved lung function despite age of patients and severity of the COPD, up to 48 weeks of treatment, also in combinatory treatment with LABA tiotropium [187,188,189,190]. A new formulation called Revefenacin is a long-acting, lung-selective muscarinic cholinergic receptor antagonist [191] and clinical trials have started in order to prove safety and efficacy of this nebulized therapy to patients (NCT02040792) [192]. Another clinical trial is assessing the therapeutic potential of an oxyhydrogen generator delivered by nebulizer to COPD patients (NCT02850185). The combination of LAMA with LABA therapies has proven effective in COPD treatment, several studies showed their efficacy with inhalers [190,193] so delivery by nebulizer may soon be standard. There are also several clinical trials comparing JN versus VMN in COPD patients with some publications already pointing to preference for VMN [194]. Interestingly, the need for improved patient-orientated personalized care [195], and better adherence to prescription guidelines for asthma patients is also noted.

Preclinical trials commonly utilize mechanical ventilation using a computer-controlled piston ventilator such as the flexiVent (SCIREQ). This ventilator combined with Aerogen Pro VMN, allows controlled aerosolization only during the inspiratory phase, and fine tuning of flow rate by monitoring duty cycle [196]. The ability to engage controlled actuation will be an essential aspect of personalized delivery to subjects where minimal wastage of a dose is essential. For example, MacLoughlin et al. (2015) utilized VMN actuation to deliver only aerosol for the middle 50% of the inspiratory phase, while delivering a plasmid-viral complex to a rat lung injury model [54].

### 5.7. Alpha (α)-1 Antitrypsin

α-1 antitrypsin deficiency (AATD) is an autosomal co-dominant condition characterized by low circulating levels of alpha (α)-1 antitrypsin (AAT) [197]. Substantial clinical benefits of intravenous AAT administration are still deliberated, and the therapy is also expensive, onerous, and time consuming to deliver. Inhalation therapy offers the opportunity for easier and more efficient delivery of AAT directly to the lungs with high potential for a more personalized commercially available inhaled AAT replacement product [198], especially as the rate of lung decline differs between patients with AATD [96]. A recent clinical trial determined that nebulization of ATT in patients with severe AATD and frequent exacerbations of COPD may have changed the pattern of episodes [199], it also found that when modifications to the use of the nebulizer were required, which led to the rate of safety events in the AAT-treated group decreasing to that of the placebo group.

Despite the widespread use of nebulizers in the management of many lung pathologies, further research is required to acquire definitive therapies. When such therapies come to pass, nebulization as a means of therapeutic delivery offers an efficient, non-invasive, and more personalized modality.

## 6. Conclusions

Nebulizers are commonly used in clinical settings for the generation of therapeutic pharmaceutical aerosols, while inhalation of these nebulized therapies provides an effective method to deliver drugs directly to the lungs particularly in mechanically ventilated patients. It is critical that new drugs, capable of being aerosolized, prove to be effective in specific disease conditions so enhanced clinical benefits arise. In order to achieve successful outcomes, it is also vital that efficient nebulization of new therapeutics occurs, along with highly effective delivery to the pulmonary epithelium of mechanically ventilated patients.

Lab-based in vitro data in conjunction with clinical in vivo knowledge provide for widespread vibrating mesh nebulizer delivery of bronchodilators such as albuterol sulfate and ipratropium bromide, anti-inflammatory agents such as budesonide and antibiotics, such as colistin and tobramycin. For emerging therapeutics, the use of vibrating mesh nebulizers should prove beneficial to ensure adequate aerosol drug delivery, however, the ultimate efficacy of these drugs in patients has yet to be established. To date, a high-quality body of evidence from clinical trials in mechanically ventilated patients to measure the efficacy of newer therapies is still to develop.

Due to the challenges presented by the delivery of drugs to the injured lungs of mechanically ventilated patients, it will be necessary to fully optimize drug and device-related factors for effective drug delivery. Therefore, as further developments of personalized therapeutics and vibrating mesh technologies emerge through careful clinical assessment, a varied range of new aerosol therapies with valuable patient outcomes such as regional pulmonary targeting, sustained bioactivity, and specific disease indications should emerge.
